# Re-visiting the ATA 2015 sonographic guidelines - who are we missing?: A retrospective review

**DOI:** 10.1186/s40463-018-0296-5

**Published:** 2018-09-03

**Authors:** D. S. Chan, K. Gong, M. G. Roskies, V. I. Forest, M. P. Hier, R. J. Payne

**Affiliations:** 1 Department of Otolaryngology Head and Neck Surgery, Jewish General Hospital, McGill University, 3755 Côte-Ste-Catherine Road, Montreal, H3T 1E2 Canada; 2 Faculty of Medicine, McGill University, Montreal, Canada

**Keywords:** Thyroid nodule, Thyroid Cancer, FNAB, Ultrasound

## Abstract

**Background:**

The American Thyroid Association published revised guidelines in 2015 on the management of differentiated thyroid cancer in adults. One of the key changes introduced in the revision proposes that diagnostic biopsy be based on ultrasound findings (i.e. size and nodule characteristics). The overall effect of these changes results in fewer nodules requiring biopsy. This study was conducted to determine if the changes to the guidelines will result in overlooked thyroid cancers, specifically malignancies with aggressive characteristics measuring between 1 and 1.49 cm.

**Methods:**

Patients (*n* = 2083) with thyroid nodules who underwent total or subtotal/hemi thyroidectomy with or without neck dissection by a single surgeon between 2006 and 2016 were retrospectively enrolled. Demographic information and nodule characteristics were collected for all patients. Ultrasonography and final pathology reports were reviewed for patients with thyroid nodules between the sizes of 1–1.49 cm (*n* = 155).

**Results:**

45% (*n* = 70) of patients with nodules between 1 and 1.49 cm were “low suspicion” nodules according to ultrasound. 47 of these nodules contained malignancies on final histopathological examination, 100% of which were of the papillary subtype. 21% (*n =* 10) of these malignant nodules demonstrated extrathyroidal extension and 34% (*n* = 16) were associated with regional metastases.

**Conclusions:**

Reliance on sonographic patterns alone could result in missed cancer diagnoses in patients with thyroid nodules measuring between 1 and 1.49 cm. Moreover, a portion of these malignancies may be associated with aggressive features. The effect of this finding on long-term outcomes is unclear.

## Background

Thyroid nodules are a common presenting problem, detectable in up to 68% of the general population [1, 2]. Recent epidemiological studies show that the incidence of true thyroid cancer diagnoses is on the rise, nearly tripling in the last thirty years [3]. Thyroid sonography is an inexpensive and noninvasive test that is performed in patients with thyroid nodules. Certain ultrasound characteristics have been well-established as predictors of malignancy, including the presence of microcalcifications, nodule hypoechogenicity, irregular nodule borders, and a taller-than-wider shape [4]. These findings are then used to stratify the risk of malignancy and aid in decision-making regarding the necessity for more invasive investigations like ultrasound fine needle aspiration (USFNA).

The American Thyroid Association (ATA) published revised guidelines for the management of differentiated thyroid cancer in 2015 [4]. One of the key changes introduced in the revision proposes that diagnostic biopsy be based on ultrasound findings (i.e. size and nodule characteristics). The diversion from recommendations made in 2009 [5] come after review of evidence that sensitivity and specificity of sonographic characteristics predictive of malignancy improve when looking at a constellation of features, rather than individual ones [6, 7]. Thus, the 2015 ATA guidelines stratify thyroid nodules based on sonographic patterns into five levels of suspicion from “benign” to “high suspicion”; recommendations for or against USFNA are then given based on nodule size. The overall effect of these changes ultimately results in fewer nodules requiring biopsy [8].

Given the imperfect sensitivities of sonographic characteristics for predicting malignancy, it is possible that the exclusive reliance on these characteristics will lead to missed diagnoses. This study was conducted to determine if the changes to the guidelines will result in overlooked thyroid cancers, specifically malignancies with aggressive characteristics measuring between 1 and 1.49 cm.

## Methods

Ethics approval was obtained from the Research Ethics Board at a single academic institution. All patient information was anonymized and stored in a password-encrypted database.

### Patient selection

Between August 2006 and June 2016, 2382 patients with thyroid nodules underwent thyroid surgery by a single surgeon at our institution. Patients who underwent total or subtotal/hemi-thyroidectomy for thyroid nodules were included. Patients without a pre-operative ultrasound report, patients undergoing completion thyroidectomies, and patients undergoing thyroid surgery for reasons other than thyroid nodules were excluded. A total of 2083 patients were included in our analyses.

### Clinical data collected

Clinical data for all patients were collected from outpatient and inpatient charts, ultrasound and pathology reports. Data collected from all patients included age, sex, number of thyroid nodules, largest nodule size and tumor type based on final histopathology. For patients with the largest nodule between 1 and 1.49 cm, pre-operative ultrasound reports were also reviewed. This group selection was based on the 2015 ATA guidelines, which categorize thyroid nodules first based on ultrasonography findings, and then on size. For nodules of “low” suspicion (i.e. hyperechoic, partially cystic, regular margins without microcalcifications), the guidelines recommend performing USFNA on nodules ≥1.5 cm. Given this recommendation, nodules between 1 and 1.49 cm with low suspicion ultrasound patterns represent a group of potentially missed or delayed malignancy diagnoses. Final pathology, lymph node involvement, staging and tumor status were recorded for nodules in this group to determine the proportion of small nodules that would be missed if the decision to proceed to USFNA were based on sonography patterns of the 2015 ATA guidelines alone. Note that in our group of patients with thyroid nodules between 1 and 1.49 cm, the decision to operate was based on pre-op USFNA results which reflects the 2009 ATA guidelines.

### Statistical analysis

Statistical analysis was performed using “MedCalc” version 17.4. Simple descriptive statistics including means and standard deviations for continuous variables and percentages for categorical data were used. Unpaired T-tests and chi-square tests were used to compare malignancies that were included in analysis and those that were excluded, as well as potentially missed/delayed diagnoses and detected malignancies.

## Results

A total of 2083 patient charts were retrospectively reviewed for the purposes of this study. Females comprised 80.8% of our sample and the average age was 50 years old (SD 13.8). Demographic data are summarized in Table 1. The majority of the patients were operated on for a single thyroid nodule, 1208 (58%), with the mean largest nodule size being 3.02 cm (SD 1.44).

**Table 1:** Demographic information

	*N (%)*
Total patients	2083
Females	1683 (81%)
Age	
Mean	50
Range	11–91
Total Thyroidectomy	1316 (63%)

One thousand eight hundred sixty (89.3%) of patients had a largest thyroid nodule identified on ultrasound of ≥1.5 cm in size (Table 2). On pathological examination, 625 (30%) thyroid glands contained benign histology, 793 (38%) had evidence of micropapillary carcinoma, 991 (48%) of papillary cancer, 23 (2.8%) of follicular cancer, 23 of medullary (1.1%), 1 (0.04%) anaplastic thyroid cancer and 6 (0.3%) that were classified as “Other” (Table 3). The cancers classified as “Other” included lymphoma, metastatic colorectal carcinoma, neuroendocrine carcinoma, sclerosing mucoepidermoid carcinoma and insular carcinoma. It is important to note, for these results, that some thyroid glands operated on had more than one type of cancer histology found in the pathological specimen, that micropapillary was considered separate from papillary cancer, and that the size of the nodule was based on ultrasound reports.

**Table 2:** Largest nodule size

*Largest Nodule Size*	*N (%)*
< 1 cm	44 (2%)
1–1.49 cm	179 (9%)
≥1.5 cm	1860 (89%)

**Table 3:** Distribution by largest nodule size on ultrasound and any histology identified on pathology

	*Benign*	*Micropapillary*	*Papillary*	*Medullary*	*Follicular*	*Anaplastic*	*Other*
< 1 cm	2	37	0	7	0	0	0
1–1.49 cm	34	83	108	1	1	0	0
≥1.5 cm	589	694	862	15	58	1	6
Total	625	793	991	23	59	1	6

There were 179 patients with the largest nodule size between 1 and 1.49 cm. 24 patients were excluded from further analyses based on a lack of pre-operative ultrasound report and age. Of the remaining 155 patients, 70 (45%) had nodules without hypoechogenicity or microcalcifications, making them “low suspicion” nodules, and thus would not have met criteria for biopsy using the 2015 ATA guidelines (Fig. 1).

Fourty-seven “low suspicion” nodules contained malignancy on final histopathology. 10 (21%) nodules were staged as T3 based on extra thyroidal extension (Table 4), 16 (34%) demonstrated lymph node involvement (3 with extranodal extension), 7 (15%) with lymphovascular invasion, and 1 (2%) displaying a tall cell variant histology (Table 5). Staging information is summarized in Table 6. All 47 tumours were papillary or micropapillary carcinomas on final histopathological examination. Nodules classified as “micropapillary” carcinomas were only included if the size of the largest nodule on pathology was within 2 mm of the size of the largest nodule identified on ultrasound. A micropapillary carcinoma on final pathology with a > 2 mm difference was considered an incidental finding and was excluded. This cut off was chosen as there can be discrepancies of 2 mm between thyroid ultrasound and gross pathological size of carcinomas [9].

**Table 4:** T-Staging of potentially missed malignant nodules between 1 and 1.49 cm

*T Stage*	*N (%)*
T1	36 (77%)
T2	1 (2%)
T3	10 (21%)
T4	0

**Table 5:** Aggressive features of potentially missed malignant nodules between 1 and 1.49 cm

*Aggressive Feature*	*N (%)*
Positive lymph node	16 (34%)
Extra-nodal extension	3 (6%)
Extra-thyroidal extension	10 (21%)
Lymphovascular invasion	7 (15%)
Tall Cell Variant	1 (2%)

**Table 6:** Staging of potentially missed malignant nodules between 1 and 1.49 cm

*Stage*	*N (%)*
1	34 (72%)
2	1 (2%)
3	10 (21%)
4	2 (4%)

Patient and tumor characteristics for nodules with suspicious ultrasound findings (and thus would have been “detected”) and those without (“potentially missed” nodules) were not significantly different (Table 7).

**Table 7:** Comparison of potentially missed and detected malignant nodules between 1 and 1.5 cm

	Detected *N* = 74		Potentially Missed *N* = 47		χ^2^	*p*
	*n*	*%*	*n*	*%*		
Age (years)						
< 45	31	42	20	43	0.005	0.943
≥45	43	58	27	57		
Sex						
Male	11	15	11	23	1.397	0.2372
Female	63	85	36	77		
Tumor type						
Micropapillary	19	26	4	9	7.156	0.0671
Papillary	53	72	43	92		
Medullary	1	1	0	0		
Follicular	1	1	0	0		
T stage						
1	57	77	36	77	0.755	0.8603
2	1	1	1	2		
3	15	20	10	21		
4	1	1	0	0		
Stage						
1	62	84	34	72	4.409	0.2206
2	1	1	1	2		
3	11	15	10	21		
4	0	0	2	4		
ETE						
+	16	22	10	21	0.002	0.9642
–	58	78	37	79		
LN						
+	21	72	16	34	0.431	0.5116
–	53	28	31	66		

## Discussion

This study demonstrates that the changes to the ATA 2015 guidelines regarding which thyroid nodules require USFNA will likely result in delayed or potentially missed cancer diagnoses in patients at our institution. Moreover, a number of these missed malignancies are associated with aggressive features.

Sonographic characteristics of thyroid nodules have been widely studied for their value in predicting malignancy. Reported sensitivities and specificities for individual characteristics vary widely between studies [10–12], but a recent meta-analysis of over 18,000 thyroid nodules found that hypoechogenicity has a sensitivity of 73% and specificity of 56%, whereas the sensitivity and specificity for internal calcifications were 54% and 81% respectively [13]. When ultrasonography findings are combined into patterns, as presented in the 2015 ATA Guidelines, specificity and positive predictive values increase, while sensitivities are diminished. Taken together, the combination of hypoechogenicity, microcalcification, and margin irregularity has the most predictive power, but a sensitivity between 41 and 65% [13]. However, negative predictive values are reportedly high, between 97 and 98% for varying combinations of features [13]; this is most likely due to the low rate of malignancy in this group, cited to be between 5 and 10% [4]. Given these findings then, it is unsurprising that the 2015 ATA guidelines makes only a *weak recommendation* on nodules with a low suspicion sonographic pattern, emphasizing the need for further data collection.

Small thyroid nodules between the sizes of 1-2 cm present an especially interesting diagnostic question, due to their increasing prevalence and varying clinical prognosis. It has been estimated that tumors smaller than 2 cm account for 87% of the increase in thyroid cancer incidence in the United States [14]. Although the ATA guidelines provide size cutoffs for indications to proceed with USFNA, nodule size has a somewhat unclear relationship to thyroid cancer risk. Whereas one research group found that nodules greater than 2 cm had a moderately increased risk of malignancy versus nodules less than 2 cm (15% vs. 10.9%) [15], meta-analysis found that thyroid nodule size was not an accurate predictor of thyroid cancer [12]. Moreover, the cutoff of 1.5 cm for low suspicion nodules is not based on unanimous consensus. In fact, a comparison of six different guidelines for thyroid nodules describes that, the guidelines that did not apply this size cutoff (TIRADS and Kim criteria) had similar or higher performances than those that did [16].

Justification for the higher size cutoff of low suspicion nodules relies on the finding that iso- or hyper-echoic nodules without calcification are more likely to be follicular thyroid cancers (FTC) or follicular variants of papillary thyroid cancer (PTC) [17]. It has been reported that distant metastases are rarely observed from FTC < 2 cm in diameter [18], thereby explaining why observation may be warranted when the nodule is < 1.5 cm in size. In our patient population, however, 100% of detected malignancies with low suspicion ultrasound features contained papillary carcinoma, which has earlier progression to extra-thyroidal extension and lymph node metastases as compared with FTC [18].

This study has enumerated the number of delayed or potentially missed diagnoses of thyroid malignancies in patients presenting with small low suspicion nodules. The effect on long-term patient outcomes is less clear. Since the ATA guidelines do suggest repeated ultrasounds for low suspicion nodules, it is uncertain if delaying treatment for these nodules would have any impact on patient morbidity or mortality. Recent studies have aimed at demonstrating the indolent nature of papillary thyroid cancers - especially microcarcinomas, defined as tumors 1 cm or less in size. It has been reported that only 7–8% of patients with papillary thyroid microcarcinomas show tumor enlargement at 5 and 10-year follow-ups without intervention [19, 20]. Here, despite the lack of outcome measures, we have shown that the thyroid cancers that could have potentially been missed or have had a delay in diagnosis are not only of significant size (> 1 cm), but also associated with aggressive features, such as lymph node metastasis, extranodal extension, extra-thyroidal extension and tall-cell variant histology. These features have been reported by multiple studies to be independent prognostic factors associated with poorer patient outcomes and increased disease-specific mortality [21–23]. As such, future directions of research must answer the question: what are the consequences of these delayed or potentially missed cancer diagnoses on patient survival?.

Furthermore, given the uncertainties surrounding the predictive values of sonographic findings and nodule size, clinical information obtained on history and physical exam should not be ignored when making decisions regarding clinical investigations. Accordingly, the 2015 ATA Guidelines suggest that since clinical risk factors have not been included in multivariate analyses of sonographic features and thyroid malignancy risk, USFNA can be considered at lower size cutoffs for all nodules in the presence of these risk factors [4]. Future directions of research should aim to identify patient factors that predict malignancy in otherwise unsuspicious nodules, allowing for the use of the complete clinical picture in decision making.

Limitations of this study include its retrospective nature and selection bias, as only nodules that underwent surgical removal were included in our database. However, the use of this surgical database does not overestimate the absolute number of potentially missed malignancies and thus should not affect the validity of our findings. Also, the long-term outcome of these patients is unknown. In other words, if an aggressive malignancy goes undetected for a longer period of time, is the patient’s outcome affected.

## Conclusions

This study shows that although the pattern of sonographic features associated with a thyroid nodule confers a risk of malignancy, the exclusive reliance on these features could lead to delayed or missed cancer diagnoses. Furthermore, a proportion of these missed malignancies contain aggressive features associated with poorer patient prognosis. Although the exact effect on long-term patient survival is still unclear, our data provide novel insight into a diagnostic gray-zone and demonstrate the need for further evaluation of this patient population for optimal patient care and resource management.

## Abbreviations

ATA: American Thyroid Association
FTC: Follicular Thyroid Carcinoma
PTC: Papillary Thyroid Carcinoma
SD: Standard Deviation
USFNA: Ultrasound fine needle aspiration
